# Clinical Comparison of QUANTA Flash dsDNA Chemiluminescent Immunoassay with Four Current Assays for the Detection of Anti-dsDNA Autoantibodies

**DOI:** 10.1155/2015/902821

**Published:** 2015-01-05

**Authors:** Maria Infantino, Francesca Meacci, Chelsea Bentow, Peter Martis, Maurizio Benucci, Antonella Afeltra, Amelia Rigon, Fabiola Atzeni, Piercarlo Sarzi-Puttini, Mariangela Manfredi, Michael Mahler

**Affiliations:** ^1^Immunology and Allergology Laboratory Unit, S. Giovanni di Dio Hospital, Via Torregalli 3, 50143 Florence, Italy; ^2^Inova Diagnostics, Inc., San Diego, CA 92131, USA; ^3^UO Reumatologia, Ospedale S. Giovanni di Dio, 3055 69321 Firenze, Italy; ^4^UOC Medicina Clinica e Reumatologia, Università Campus Bio-Medico, Via Álvaro del Portillo 21, Roma, Italy; ^5^UOC Reumatologia, Ospedale L. Sacco Polo Universitario, Via Giovanni Battista Grassi 74, 20157 Milano, Italy

## Abstract

*Introduction*. The objective of the present study was to compare QUANTA Flash dsDNA, a chemiluminescent immunoassay (CIA) on the BIO-FLASH, a rapid-response chemiluminescent analyzer, to three other anti-dsDNA antibody assays and to *Crithidia luciliae* indirect immunofluorescence test (CLIFT). *Methods*. In the first part of the study, 161 samples, 61 from patients suffering from systemic lupus erythematosus (SLE) and 100 from a disease control group, were tested by QUANTA Flash dsDNA CIA, QUANTA Lite dsDNA SC ELISA, BioPlex 2200 multiplex flow immunoassay (MFI), ImmuLisa dsDNA ELISA, and NOVA Lite CLIFT. A second cohort of 69 SLE patients was then tested by QUANTA Flash dsDNA and CLIFT to expand the study. *Results*. The overall qualitative agreements varied between 77.0% (NOVA Lite CLIFT versus QUANTA Lite) and 89.4% (ImmuLisa versus NOVA Lite CLIFT). The clinical sensitivities for the anti-dsDNA antibody tests varied from 8.2% (NOVA Lite CLIFT) to 54.1% (QUANTA Lite), while the clinical specificities varied from 88.0% (BioPlex 2200) to 100.0% (NOVA Lite CLIFT). Good correlation was found between QUANTA Flash dsDNA and NOVA Lite CLIFT. *Conclusion*. Significant variations among dsDNA methods were observed. QUANTA Flash dsDNA provides a good combination of sensitivity and specificity for the diagnosis of SLE and good agreement to CLIFT.

## 1. Introduction

The detection of anti-dsDNA autoantibodies represents a hallmark in the diagnosis of systemic lupus erythematosus (SLE) [1] and these antibodies are part of the classification criteria for SLE [2, 3]. Additionally, anti-dsDNA antibodies measured by certain assays have been reported to correlate with disease activity and lupus nephritis. Despite the fact that several assay systems have been developed during the last decades, controversies persist on which assay is the best test to measure anti-dsDNA antibodies [4, 5]. Although the Farr assay has been considered the “gold standard” for the detection of anti-dsDNA antibodies in many laboratories [4, 6, 7], radioimmunoassays in general have recently been falling into disuse in routine practice based on several factors, including the radioactive nature of the assay. Therefore, it is important to evaluate new fully automated technologies which have recently become available as viable alternatives to Farr assay. The objective of the present study was to compare QUANTA Flash dsDNA, a chemiluminescent immunoassay (CIA) on the BIO-FLASH, a rapid-response chemiluminescent analyzer, to three other anti-dsDNA antibody assays and to* Crithidia luciliae* indirect immunofluorescence test (CLIFT).

## 2. Materials and Methods

### 2.1. Sera

In the first part of the study, 161 samples, 61 from patients suffering from SLE and 100 controls, including Hashimoto's thyroiditis (*n* = 40), rheumatoid arthritis (*n* = 40), and blood donors (*n* = 20) from apparently healthy individuals, were tested for anti-dsDNA IgG by QUANTA Flash dsDNA CIA (Inova Diagnostics, San Diego, CA, USA), QUANTA Lite dsDNA SC ELISA (Inova Diagnostics Inc.), BioPlex 2200 MFI (Bio-Rad, USA), ImmuLisa dsDNA ELISA (Immco Diagnostics, Buffalo, NY, USA), and NOVA Lite CLIFT (Inova Diagnostics). A second cohort of 69 SLE patients was tested by QUANTA Flash dsDNA CIA and NOVA Lite CLIFT to expand the first part of the study for these methods. The diagnoses were established as described in a previous paper before or, if applicable, according to the standard disease criteria [8].

This study meets and is in compliance with all ethical standards in medicine, and informed consent was obtained from all patients according to the Declaration of Helsinki.

### 2.2. QUANTA Flash dsDNA

The QUANTA Flash dsDNA (Inova Diagnostics Inc.) assay is a novel CIA that is used on the BIO-FLASH instrument (Biokit s.a., Barcelona, Spain), fitted with a luminometer, as well as the hardware and liquid handling accessories necessary to fully automate the assay. The principle of the BIO-FLASH system has recently been described [9, 10]. The QUANTA Flash assay for this study was developed using synthetic dsDNA (see Table 1) coupled to the surface of paramagnetic beads. Prior to use, the lyophilized beads are resuspended using the resuspension buffer. A patient serum sample is prediluted with the BIO-FLASH sample buffer in a small disposable plastic cuvette. Small amounts of the diluted patient serum, the beads, and the assay buffer are all combined into a second cuvette, mixed, and then incubated for 9.5 minutes at 37°C. The magnetized beads are sedimented using a strong magnet in the washing station and washed several times followed by addition of isoluminol conjugated anti-human IgG and again incubated for 9.5 minutes at 37°C. The magnetized beads are sedimented and washed repeatedly. The isoluminol conjugate is oxidized when sodium hydroxide solution and peroxide solutions (triggers) are added to the cuvette, and the flash of light produced from this reaction is measured as relative light units (RLUs) by the BIO-FLASH optical system. The RLUs are proportional to the amount of isoluminol conjugate that is bound to the human IgG, which is in turn proportional to the amount of autoantibodies bound to the antigen on the beads.

### 2.3. BioPlex 2200

BioPlex 2200 (Bio-Rad, Hercules, CA) system is an automated analyzer that uses multiplex bead technology (Luminex, Austin, TX, USA) to simultaneously detect antibodies to several antigens in a single tube. The BioPlex 2200 ANA Screen is intended for the qualitative screening of ANA, the quantitative detection of antibody to dsDNA, and the semiquantitative detection of ten separate antibodies (Chromatin, Ribosomal P, SS-A, SS-B, Sm, SmRNP, RNP, Scl-70, Jo-1, and Centromere B) [11, 12] in human serum and/or EDTA or heparinized plasma. The test system is used as an aid in the diagnosis of SARD. Characteristics of the assay are summarized in Table 1.

### 2.4. ELISAs (ImmuLisa dsDNA ELISA and QUANTA Lite dsDNA SC ELISA)

Both the ImmuLisa dsDNA (Immco Diagnostics) and QUANTA Lite dsDNA SC (Inova Diagnostics Inc.) are enzyme linked immunosorbent assays (ELISA) for the quantitative or semiquantitative detection of IgG specific for dsDNA in human serum as an aid in the diagnosis of SLE in conjunction with other laboratory and clinical findings. Both assays were performed at Inova Diagnostics according to the direction insert. Characteristics of the assays are summarized in Table 1.

### 2.5. NOVA Lite dsDNA* Crithidia luciliae* (CLIFT)

NOVA Lite dsDNA CLIFT (Inova Diagnostics Inc.) is an indirect immunofluorescence (IIF) assay for the screening and titration based determination of anti-dsDNA antibodies in human serum. The NOVA Lite dsDNA CLIFT employs the hemoflagellate* Crithidia luciliae* as a substrate. This single-cell organism possesses a giant mitochondrion containing a highly condensed mass of circular dsDNA. The assay was performed by a single operator using an LED microscope at Inova Diagnostics according to the direction insert. CLIFT results were graded from 0 to 4 according to the intensity (see also direction insert of the kit); 4 is brilliant apple green fluorescence; 3 is bright apple green fluorescence; 2 is clearly distinguishable positive fluorescence; 1 is lowest specific fluorescence that enables the kinetoplast staining to be clearly differentiated from the background fluorescence; 0 is negative. Characteristics of the assay are summarized in Table 1.

### 2.6. Precision and Linearity Studies

Precision and linearity of the QUANTA Flash dsDNA CIA were verified by performing the required testing according to the Clinical and Laboratory Standards Institute (CLSI) guidelines. For the precision study, the within-run, between-day, between-run, and total precision were determined by running two aliquots of the precision samples twice a day in random order, with a minimum of 2 hours between each run. The samples were run on the same instrument for each assay and repeated for at least 20 days, according to CLSI guideline EP5-A2. Linearity testing was performed according to CLSI guideline EP6-A (volume 23, number 16), which involved diluting several high titer sera in a serial dilution scheme to span the analytical measuring range (AMR) for each assay.

### 2.7. Statistical Analyses

The data were statistically evaluated using the Analyse-it software (version 1.62; Analyse-it Software, Ltd., Leeds, UK). Spearman's correlation and Cohen's* kappa* agreement test were carried out to analyze the agreement between portions and *P* values < 0.05 were considered significant. Receiver operating characteristics (ROC) analysis was used to analyze the discriminatory ability of different immunoassays. Cluster analysis was used to illustrate the relationship between different assays [13] and to display the reactivity pattern of the patients. Hierarchical clustering was performed using average linkage clustering where patient correlation was performed uncentered and the reactivities were uncentered. For the assays having an equivocal range, all data was analyzed using the applied cut-offs listed in Table 1, where the equivocal results were considered positive.

## 3. Results

### 3.1. Precision and Linearity

For the precision testing of QUANTA Flash dsDNA CIA, seven samples ranging from 27.3 to 402.8 IU/mL were tested. For all samples, the within-run varied between 4.7 and 6.9%, the between-day varied between 2.3 and 4.0%, and the between-run varied between 0.0 and 4.9%. The total precision for the assay varied between 6.6 and 8.2%. The linearity study showed linearity of the QUANTA Flash dsDNA CIA from 7.8 to 683.8 IU/mL with a slope of 1.06 (95% confidence interval 1.04–1.08), a *Y*-intercept of −2.0 (−6.9–2.8), and an *R* ^2^ of 0.99. QUANTA Flash dsDNA CIA demonstrated linearity over the entire AMR.

### 3.2. Qualitative and Quantitative Agreements between dsDNA Methods

The overall qualitative agreements varied between 77.0% (NOVA Lite CLIFT versus QUANTA Lite) and 89.4% (ImmuLisa versus NOVA Lite CLIFT).* Kappa* agreements were low ranging from 0.17 to 0.42 (QUANTA Lite and QUANTA Flash versus BioPlex). Total percent agreement,* kappa* values, and Spearman's* rho* between dsDNA methods can be found in Table 2 (Spearman correlation graphs between dsDNA methods are not shown).

### 3.3. Clinical Performance of Five Anti-dsDNA Assays

**Figure 1:**
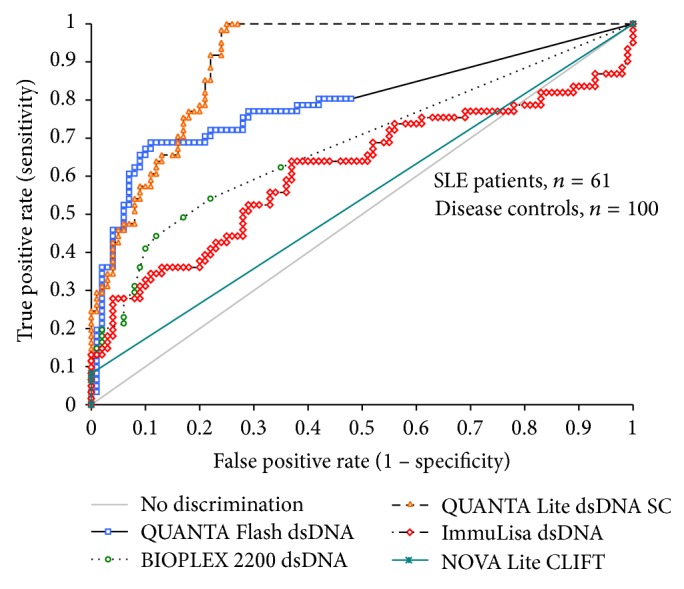
Comparison of different assays for the detection of anti-dsDNA antibodies using receiver operating characteristics (ROC) analysis. The ROC curves show the discrimination between SLE patients (*n* = 61) and controls (*n* = 100) using different assays. Note: the NOVA Lite CLIFT assay is a semiquantitative assay (grades 0 to 4 were given by operator).

The clinical sensitivities at the recommended cut-off were 8.2% (95% confidence interval, CI 2.7–18.1%) for NOVA Lite CLIFT, 26.2% (95% CI 15.8–39.1%) for ImmuLisa, 39.3% (95% CI 27.2–52.7%) for QUANTA Flash, 44.3% (95% CI 31.5–57.6%) for BioPlex, and 54.1% (95% CI 40.8–66.9) for QUANTA Lite. The clinical specificities at the recommended cut-off were 100.0% (95% CI 96.4–100.0%) for NOVA Lite CLIFT, 96.0% (95% CI 90.1–98.9%) for ImmuLisa, 96.0% (95% CI 90.1–98.9%) for QUANTA Flash, 88.0% (95% CI 80.0–93.6%) for BioPlex, and 91.0% (95% CI 83.6–95.8%) for QUANTA Lite. When the sensitivities were compared at a specificity of 94.0%, the values ranged between 8.2% (95% CI 2.7–18.1%) for CLIFT and 52.5% (95% CI 39.3–65.4%) for QUANTA Flash dsDNA CIA. Sensitivities and specificities as well as positive and negative likelihoods ratios (LR) and area under the curve (AUC) values for all assays are shown in Table 3. The odds ratio (LR+/LR−) for dsDNA methods varied between 5.9 (BioPlex) and 15.6 (QUANTA Flash). Comparative ROC curve analysis among SLE patients (*n* = 61) and disease controls (*n* = 100) for five anti-dsDNA antibody assays showed significantly different AUC values. QUANTA Flash and QUANTA Lite had the highest AUC values which were significantly higher (*P* < 0.05) than the AUC values of the other assays (see Figure 1).

### 3.4. Cluster Analysis

**Figure 2:**
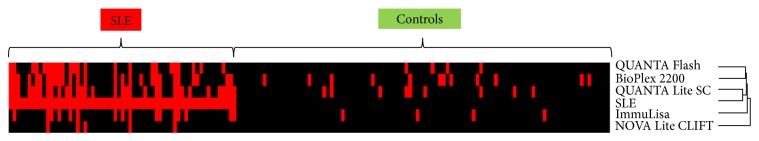
Supervised cluster analysis of the results. Supervised centered cluster analysis according to disease cohort (SLE versus controls) is shown. The dendrogram shows the QUANTA Lite dsDNA SC clusters closest to the diagnosis of SLE followed by QUANTA Flash and BioPlex 2200. SLE: systemic lupus erythematosus.

To illustrate the reactivity of various assays in relation to the diagnosis, we performed a cluster analysis. The cluster analysis shows that the majority of SLE patients have multiple positive results (Figure 2). Some of the controls also show positive results by different methods. The dendrogram shows the QUANTA Lite dsDNA SC clusters closest to the diagnosis of SLE followed by QUANTA Flash and BioPlex 2200.

### 3.5. Comparison of Anti-dsDNA Assays with CLIFT and Extended Clinical Evaluation for QUANTA Flash dsDNA

**Figure 3:**
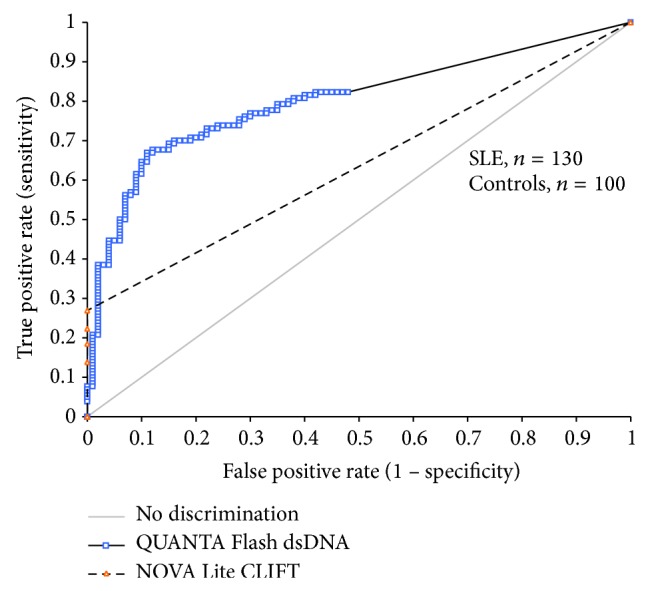
Comparison of NOVA Lite CLIFT and QUANTA Flash dsDNA for the detection of anti-dsDNA antibodies using receiver operating characteristics (ROC) analysis. The ROC curves show the discrimination between SLE patients (*n* = 130) and controls (*n* = 100). Note: the NOVA Lite CLIFT is a semiquantitative assay (grades 0 to 4 were given by operator).

**Figure 4:**
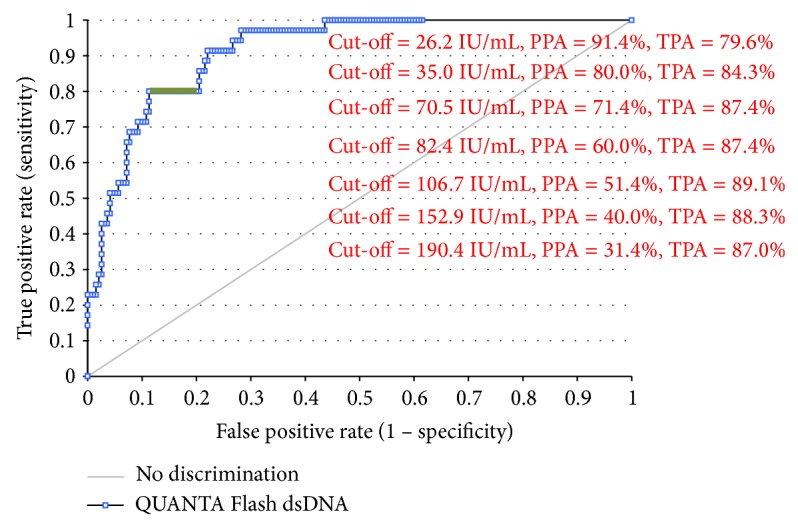
Receiver operating characteristics (ROC) analysis of QUANTA Flash dsDNA compared to CLIFT positive (*n* = 35) and negative (*n* = 195) samples. Different cut-offs are shown to demonstrate the optimal agreements. Note: equivocal range determined by the manufacturer is along the 80.0% positive agreement (green highlight). PPA: positive percent agreement; TPA: total percent agreement.

The CLIFT is often used as a confirmatory assay. Consequently, we used the CLIFT results (positive versus negative) as the reference and analyzed the utility of the other assays to match the sensitivity and specificity of the CLIFT. All CLIFT positive samples were also positive by all four methods, except for one sample that was missed by the ImmuLisa. ROC analysis (ROC curve not shown) of the four anti-dsDNA antibody assays compared to CLIFT resulted in AUC values of 0.93 (95% CI 0.85–1.00) for BioPlex, 0.94 (95% CI 0.88–1.00) for ImmuLisa, 0.95 (95% CI 0.91–0.99) for QUANTA Lite, and 0.97 (95% CI 0.94–0.99) for QUANTA Flash. A cut-off of 106.7 IU/mL for QUANTA Flash dsDNA would result in an optimal agreement to CLIFT, with a positive agreement of 100.0% (95% CI 47.8–100.0%), negative agreement of 95.5% (95% CI 91.0–98.2%), total agreement of 95.7% (95% CI 91.2–98.2%), and* kappa* = 0.57 (95% CI 0.29–0.85). To further validate the good agreement between QUANTA Flash dsDNA and CLIFT, additional SLE patients (*n* = 69, Inova Diagnostics serum bank) tested by both methods were added to the study population. ROC analysis of the clinical performance for NOVA Lite CLIFT and QUANTA Flash dsDNA among the new study population (SLE = 130, controls = 100) can be found in Figure 3. ROC analysis for the agreement of QUANTA Flash dsDNA results to NOVA Lite CLIFT results can be found in Figure 4.

## 4. Discussion

Anti-dsDNA antibodies are a hallmark in the diagnosis of SLE and are part of the classification criteria [2]. During the last decades, several novel technologies have been developed for anti-dsDNA antibody detection including the conventional ELISA and, more recently, line immunoassays (LIA), CIA, and multiplex assays [11, 12]. As ELISAs are only moderately fast with assay times between 1.5 and 3 hours, the focus has lately shifted towards a decrease in assay time and ease of use. Despite significant technological advances, controversies persist on the method of choice for the detection of these antibodies [4, 14]. The BioPlex 2200 dsDNA assay has been compared to other assays (Farr and Farrzyme) in a recent study [15]. Additionally, several studies used the BioPlex 2200 dsDNA assay as the only method for the detection of anti-dsDNA antibodies [16, 17]. The sensitivity in our cohort (44.3%) was very similar to the data (40%) presented by Op De Beéck et al. [16] but significantly lower than previous results [18]. The CLIFT assay is an IIF test based on the hemoflagellate* Crithidia luciliae* as the substrate. Commercial assays are offered by various companies. Various commercial ELISA tests are available through numerous companies. Although based on the same technology, poor agreement can be observed, as seen in previous studies [14, 15, 19, 20]. Similar to the sensitivity observed for BioPlex 2200 dsDNA in this study, the sensitivity in SLE found for the two ELISAs was lower than previous studies [21, 22], indicating that the SLE sample population for this study could be the reason. Lastly, the Farr assay has been considered as the “gold standard” for the detection of anti-dsDNA antibodies [4, 6, 7]. However, not in all studies, the Farr assay was superior to other methods for the detection of anti-dsDNA antibodies and the discrimination of SLE and controls [14]. Additionally, in many countries, laboratories are switching from the Farr assay to fully automated solutions, which is largely due to the radioactive nature of the Farr assay [4, 5]. Therefore, considering that newer fully automated technologies are available, it is important to evaluate these methods as viable alternatives as the Farr assay becomes less popular. Some studies also investigated the impact of the antigen source on the performance of anti-dsDNA antibody assays. Since this study compares not only different technologies but also methods with different antigen sources, it is unclear if antigen source is a contributor to the variability in the results. This is the first study on QUANTA Flash dsDNA, a novel CIA for detection of anti-dsDNA antibodies. Like other assays on the BIO-FLASH [9, 23, 24], the QUANTA Flash dsDNA CIA delivers quantitative results with high precision and linearity in as little as 30 minutes. The QUANTA Flash dsDNA CIA showed lower sensitivity compared to the QUANTA Lite dsDNA ELISA (39.3% versus 54.1%) but had a higher specificity (96.0% versus 91.0%) and a higher odds ratio compared to the other methods. By setting the specificity to the clinically relevant level of 94.0% for all dsDNA methods, QUANTA Flash dsDNA CIA achieved the highest sensitivity (52.5%). Since the CLIFT assay is often used as a confirmatory test for the detection of anti-dsDNA antibodies, we aimed to analyze the possibility to use higher cut-off values for QUANTA Flash dsDNA to increase the agreement to CLIFT. Using a cut-off of 106.7 IU/mL, the agreement increased to 95.7%. The extended study between QUANTA Flash dsDNA CIA and NOVA Lite CLIFT with additional SLE samples resulted in good agreement between methods around the manufacturer's cut-off for QUANTA Flash dsDNA. Figure 4 displays the agreement of QUANTA Flash dsDNA to CLIFT at various cut-offs, which demonstrates the potential to match the samples positive by CLIFT. This finding is promising that, using the QUANTA Flash dsDNA, confirmation testing might become unnecessary. However, future studies are needed to confirm this observation. A limitation of our study is the number of SLE patients and controls, but the strength of the study is the number of technologies analyzed. However, despite the small cohort of patients, we found significant differences between the assays and confirmed the lack of standardization [19, 20]. Another shortcoming is the exclusion of the Farr assay from our study due to the lack of access to the technology. At our facility, assays with radioactive materials cannot be performed and the sample volumes were too low to send out samples for testing. On the other hand, we included five methods for the detection of anti-dsDNA antibodies. Further studies with larger cohorts are needed to contribute to the understanding of how anti-dsDNA antibody assays perform and correlate.

## 5. Conclusion

Significant variations among anti-dsDNA antibody assays were observed confirming the lack of standardization. QUANTA Flash dsDNA CIA provides an optimal combination of sensitivity and specificity for the diagnosis of SLE. Future studies with larger sample populations are needed to confirm the clinical performance and agreement to CLIFT.

## Figures and Tables

**Table 1:** Characteristics of the anti-dsDNA antibody assays used in this study.

Characteristic	QUANTA Flash dsDNA	QUANTA Lite dsDNA SC	BioPlex 2200 dsDNA	ImmuLisa dsDNA	NOVA Lite dsDNA
Manufacturer	Inova Diagnostics	Inova Diagnostics	Bio-Rad	Immco Diagnostics	Inova Diagnostics
Technology	CIA	ELISA	ALBIA	ELISA	CLIFT
Assay timer	30 min	90 min	45 min	90 min	60 min
Detection	Quantitative	Quantitative	Quantitative	Semi-quantitative	Semi-quantitative
Analytical measuring range	9.8–666.9 IU/mL	12.3–1000 IU/mL	1–300 IU/mL	13.6–450 IU/mL	N/A
Cut-off value (ranges)	9.8–35 negative 35–45 equivocal >45 positive	12.3–30 negative 30–75 equivocal >75 positive	1–5 negative 5–9 indeterminate >9 positive	13.6–50 negative 50–60 borderline >60 positive	N/A
Cut-off value applied	≥35 IU/mL	≥30 IU/mL	≥5 IU/mL	≥50 IU/mL	N/A
Antigen source	Synthetic dsDNA	Native calf thymus	Synthetic dsDNA	Purified native dsDNA	*Crithidia luciliae* substrate

CIA: chemiluminescent immunoassay; ELISA: enzyme linked immunosorbent assay; ALBIA: addressable laser bead immunoassay; CLIFT: *Crithidia luciliae* indirect immunofluorescence test.

**Table 2:** Qualitative and quantitative agreements between anti-dsDNA antibody assays.

	NOVA Lite dsDNA CLIFT	QUANTA Flash dsDNA	QUANTA Lite dsDNA SC	BioPlex 2200 dsDNA	ImmuLisa dsDNA
QUANTA Flash dsDNA	85.7 (79.3–90.7) *kappa* = 0.26 (0.08–0.45)		78.9 (71.8–84.9) *kappa* = 0.39 (0.22–0.55)	80.7 (73.8–86.5) *kappa* = 0.42 (0.25–0.59)	83.9 (77.2–89.2) *kappa* = 0.37 (0.17–0.56)
	N/A^*^		*rho* = 0.58 (0.46–0.67) *P* < 0.0001	*rho* = 0.46 (0.33–0.57) *P* < 0.0001	*rho* = 0.34 (0.19–0.47) *P* < 0.0001

QUANTA Lite dsDNA SC	77.0 (69.7–83.3) *kappa* = 0.17 (0.04–0.30)	78.9 (71.8–84.9) *kappa* = 0.39 (0.22–0.55)		78.3 (71.1–84.4) *kappa* = 0.42 (0.26–0.58)	80.1 (73.1–86.0) *kappa* = 0.38 (0.22–0.54)
	N/A^*^	*rho* = 0.58 (0.46–0.67) *P* < 0.0001		*rho* = 0.43 (0.30–0.55) *P* < 0.0001	*rho* = 0.39 (0.25–0.51) *P* < 0.0001

BioPlex 2200 dsDNA	78.9 (71.8–84.9) *kappa* = 0.18 (0.04–0.32)	80.7 (73.8–86.5) *kappa* = 0.42 (0.25–0.59)	78.3 (71.1–84.4) *kappa* = 0.42 (0.26–0.58)		80.7 (73.8–86.5) *kappa* = 0.37 (0.20–0.54)
	N/A^*^	*rho* = 0.46 (0.33–0.57) *P* < 0.0001	*rho* = 0.43 (0.30–0.55) *P* < 0.0001		*rho* = 0.30 (0.16–0.44) *P* < 0.0001

ImmuLisa dsDNA	89.4% (83.6–93.7) *kappa* = 0.28 (0.05–0.51)	83.9 (77.2–89.2) *kappa* = 0.37 (0.17–0.56)	80.1 (73.1–86.0) *kappa* = 0.38 (0.22–0.54)	80.7 (73.8–86.5 ) *kappa* = 0.37 (0.20–0.54)	
	N/A^*^	*rho* = 0.34 (0.19–0.47) *P* < 0.0001	*rho* = 0.39 (0.25–0.51) *P* < 0.0001	*rho* = 0.30 (0.16–0.44) *P* < 0.0001	

Note: total qualitative agreements are given in percent, followed by kappa statistics and Spearman's rho values (95% confidence intervals are provided in the parentheses). ^*^NOVA Lite CLIFT was excluded from the analysis since it is a semiquantitative assay where grading values (0 to 4) are given by the operator.

**Table 3:** Clinical performance characteristics for anti-dsDNA antibody assays.

	QUANTA Flash dsDNA	QUANTA Lite dsDNA SC	BioPlex 2200 dsDNA	ImmuLisa dsDNA	NOVA Lite dsDNA
Manufacturer's cut-off used, where equivocal results are considered positive	≥35 IU/mL	≥30 IU/mL	≥5 IU/mL	≥50 IU/mL	N/A
Sensitivity in SLE% (95% CI)	39.3 (27.2–52.7)	54.1 (40.8–66.9)	44.3 (31.5–57.6)	26.2 (15.8–39.1)	8.2 (2.7–18.1)
Specificity % (95% CI)	96.0 (90.1–98.9)	91.0 (83.6–95.8)	88.0 (80.0–93.6)	96.0 (90.1–98.9)	100.0 (96.4–100.0)
LR+	9.84	6.01	3.69	6.56	+*∞*
LR−	0.63	0.50	0.63	0.77	0.92
Odds ratio	15.6	12.0	5.9	8.5	N/A
AUC (95% CI)	0.79 (0.72–0.87)	0.90 (0.86–0.95)	0.68 (0.60–0.77)	0.61 (0.52–0.71)	0.54 (0.51–0.58)
Cut-off used at 94.0% specificity	≥27.5 IU/mL	≥49.6 IU/mL	≥12 IU/mL	≥41.9 IU/mL	N/A
Sensitivity in SLE% at 94.0% specificity (95% CI)	52.5 (39.3–65.4)	45.9 (33.1–59.2)	21.3 (11.9–33.7)	27.9 (17.1–40.8)	N/A

AUC: area under the curve; LR: likelihood ratio; CI: confidence interval.

## Abbreviations

AUC: Area under the curve
AMR: Analytical measuring range
CIA: Chemiluminescent immunoassay
CLIFT: * Crithidia luciliae* indirect immunofluorescence test
CTD: Connective tissue disease
ELISA: Enzyme-linked immunosorbent assay
LIA: Line immunoassays
MFI: Multiplex flow immunoassay
ROC: Receiver-operating characteristics
SARD: Systemic autoimmune rheumatic disease
SLE: Systemic lupus erythematosus.

## Highlights

- Significant variations among dsDNA methods were observed.
- QUANTA Flash dsDNA showed good analytical and clinical performance in this study.
- Standardization of anti-dsDNA assays still requires improvements.
